# Pattern of Ocular Injuries Following Road Traffic Accidents Seen at a Tertiary Eye Hospital

**DOI:** 10.7759/cureus.59126

**Published:** 2024-04-27

**Authors:** Takashi Ono, Takuya Iwasaki, Yosai Mori, Ryohei Nejima, Takashi Miyai, Makoto Aihara, Kazunori Miyata

**Affiliations:** 1 Ophthalmology, University of Tokyo, Tokyo, JPN; 2 Ophthalmology, Miyata Eye Hospital, Miyakonojo, JPN

**Keywords:** orbital fracture, intraocular pressure, corrected-distance visual acuity, corneal injury, clinical outcomes

## Abstract

Introduction: Traffic trauma can lead to ocular damage. Open globe injuries usually have a poor prognosis, which can be ameliorated by prompt diagnosis and appropriate treatment. Nonetheless, few studies have focused on the visual outcomes of patients following traffic accidents. In this study, we aimed to examine the characteristics and prognosis of ocular complications in patients following traffic accidents at a specialized tertiary eye hospital.

Methods: We classified 44 patients from traffic accidents (88 eyes) into groups with equal or better (*better group*) and worse (*worse group*) corrected-distance visual acuity than a logarithm of the minimum angle of resolution 0 at the initial presentation. Final corrected-distance visual acuity, intraocular pressure, corneal injury, presence of traumatic cataracts, and treatment were compared between the groups. In addition, a multivariate linear regression analysis was performed to identify factors associated with the final visual acuity.

Results: Globe contusion, orbital blowout fracture, traumatic iritis, and trochlear nerve palsy were observed in 14.8%, 3.4%, 2.3%, and 2.3% of the patients, respectively. Topical instillation and ophthalmological treatment/surgery were performed in 17.0% and 9.1% of the patients, respectively. The *better group* (68 eyes) had significantly better final visual acuity than the *worse group* (20 eyes) (*P *< 0.001). However, there was no between-group difference in demographic characteristics. Multivariate analysis demonstrated that there was a significant correlation between the initial and final visual acuities (*P *< 0.001).

Conclusions: Assessing visual acuity at the initial presentation is crucial for predicting the final visual acuity. Our findings will help to inform ophthalmologists aiming to improve the prognosis and treatment of ocular trauma in patients following traffic accidents.

## Introduction

Road traffic accidents can adversely affect numerous body organs and can be fatal. Global efforts are yet to successfully reduce the occurrence of road traffic deaths, as indicated by the Global Status Reports on Road Safety of the World Health Organization [1]; therefore, this remains a global concern [2,3]. The ocular effects of traffic trauma involve various ocular pathologies, including open globe injuries, optic nerve canal fractures, and blowout fractures [4-6]. Among them, an open globe injury generally results in poor prognosis; accordingly, it requires prompt diagnosis and appropriate treatment [7-11]. Therefore, it is important to elucidate the prognosis and proper treatment of ocular trauma to inform ophthalmologists managing patients from traffic accidents. Studies have focused on the prognosis following whole ocular trauma or open globe injury, with relatively few studies focusing on visual outcomes in patients from traffic accidents [12-16]. Ocular trauma can cause anterior segment complications, including corneal abnormalities, intraocular lens dislocations, and traumatic cataracts [17]. In clinical settings, many patients from traffic accidents present to ophthalmologists at tertiary eye hospitals. However, most studies have been performed from the perspective of emergency medicine, with few studies investigating the interventions or clinical outcomes related to the final visual acuity in patients with traffic-related injuries. Accordingly, we aimed to classify the pattern of ocular injuries following road traffic accidents, examine the ocular prognosis, and identify factors related to final visual acuity in patients seen at a tertiary eye hospital.

## Materials and methods

Patient consent and ethical disclosure

This clinical study was approved by the Institutional Review Board of Miyata Eye Hospital (Identifier: CS-383-013) and performed in accordance with the Declaration of Helsinki. Informed consent was obtained through an opt-out procedure based on ethics committee documents. For children, adults with parental authority were considered their representatives. The requirement for written informed consent was waived, given the choice of an opt-out procedure.

Patients and study design

This retrospective, observational clinical study included patients who visited Miyata Eye Hospital (Miyazaki, Japan) between 2015 and 2022 with complaints of symptoms following a traffic accident. Patients with insufficient medical information in the medical records were excluded. We retrospectively collected the following information from the medical records: age, sex, cause of accident, corrected-distance visual acuity (CDVA) at the initial and final presentation, intraocular pressure (IOP), treatment, observation period, state of the lens, corneal injury, and presence of traumatic cataracts. CDVA was measured on a decimal scale and converted to the logarithm of the minimum angle of resolution (logMAR) for statistical analyses. Visual acuity of hand motion was converted as previously described [18]. Patients were classified into those with CDVA ≤ logMAR 0 (better group) and those with CDVA > logMAR 0 (worse group) at the initial presentation. Furthermore, we evaluated the degree of ocular trauma based on the Ocular Trauma Score as previously reported [19]. We performed between-group comparisons for each item. Multiple regression analysis was performed to identify factors related to the final visual acuity, with the final CDVA as the objective variable and age, initial CDVA, days from injury to the initial visit, and availability of referral letters as the explanatory variables.

Statistical analyses

All statistical analyses were performed using GraphPad Prism (GraphPad Software, San Diego, CA, USA). The Mann-Whitney U test was used for between-group comparisons of the CDVA and IOP at the initial and last presentations, days from injury to the visit, and observation periods. Fisher’s exact test was used for between-group comparisons of sex, cause of accident, availability of referral letters, state of lenses, traumatic cataract, corneal injury, and treatment. A multivariate regression analysis was performed using the least-squares method. Statistical significance was set at P < 0.05.

## Results

**Figure 1:**
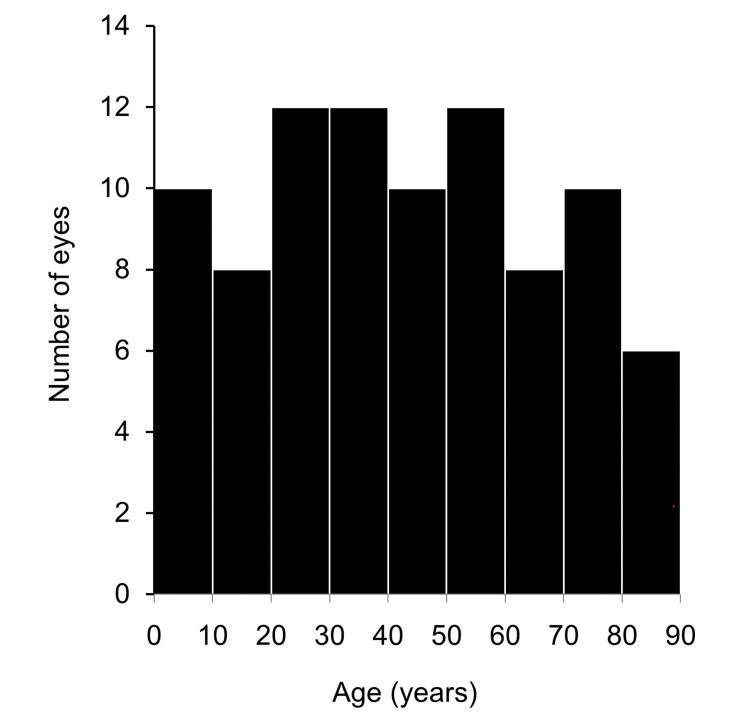
Age distribution of the included patients Most of the patients included in the study were in their 20s, 30s, and 50s.

This study included 88 eyes of 44 patients (mean age, 42.8 ± 24.2 years). Between them, 68 eyes and 20 eyes were in the better group (mean age, 42.6 ± 20.8 years) and the worse group (mean age, 43.5 ± 34.0 years), respectively. The mean duration from injury to the initial visit was 21.3 ± 38.0 days. Table 1 summarizes patient background characteristics. There were no between-group differences in sex, age, availability of referral letters from other hospitals, days from injury to visit, observation periods, and state of lenses (phakic eyes or intraocular lenses). The better group showed a significantly better initial CDVA than the worse group (Table 1, P < 0.001). Moreover, the better group had significantly higher IOP values than the worse group. However, the IOP in both groups was <20 mmHg, which was within the normal range. Figure 1 presents the age distribution of the patients. Most of the patients were in their 20s, 30s, and 50s. However, the patients were in various age groups. 

**Table 1:** Demographic characteristics of the included patients The data are presented as N (%) or mean ± standard deviation. Statistical significance was set at P < 0.05.

	All	Better group	Worse group	P value
Number of eyes (eyes)	88	68	20	-
Number of patients (patients)	44	34	10	-
Sex (male/female)	38/50	28/40	10/10	0.484
Age (years)	42.8 ± 24.2	42.6 ± 20.8	43.5 ± 34.0	0.895
Referral letter from another hospital, n (%)	34 (63.0%)	29 (42.6%)	5 (25.0%)	0.154
Cause of accident (car/motorcycle)	86/2	66/2	20/0	0.999
Days from injury to the visit (days)	21.3 ± 38.0	21.4 ± 40.6	20.4 ± 24.6	0.944
Observation periods (days)	434.7 ± 583.4	471.0 ± 603.3	271.4 ± 483.6	0.388
Initial CDVA (logMAR)	-0.051 ± 0.242	-0.114 ± 0.073	0.256 ± 0.464	< 0.001
Initial intraocular pressure (mmHg)	14.3 ± 4.1	15.0 ± 3.6	11.4 ± 4.8	0.0013
Phakic eyes, n, (%)	79 (89.8%)	62 (91.2%)	17 (85.0%)	0.423

The most frequent ocular diagnosis was globe contusion (13 eyes, 14.8%) (Table 2), followed by dry eye (four eyes, 4.5%), orbital blowout fracture (three eyes, 3.4%), and subconjunctival hemorrhage (three eyes, 3.4%). The cases of nerve damage included trochlear nerve palsy (two eyes, 2.3%), abducens nerve palsy (one eye, 1.1%), facial nerve palsy (one eye, 1.1%), oculomotor nerve palsy (one eye, 1.1%), and traumatic optic neuropathy (one eye, 1.1%). Contrastingly, the ophthalmological assessment did not reveal abnormalities in 38 (43.2%) eyes.

**Table 2:** Ocular diagnoses of the patients following traffic accidents The data are presented as N (%).

Diagnosis	Eyes n, (%)
No abnormality	38 (43.2%)
Contusion of the globe	13 (14.8%)
Dry eye	4 (4.5%)
Orbital blowout fracture	3 (3.4%)
Subconjunctival hemorrhage	3 (3.4%)
Accommodative spasm	2 (2.3%)
Bitemporal hemianopsia	2 (2.3%)
Conjunctival foreign body	2 (2.3%)
Convergence insufficiency	2 (2.3%)
Lacrimation	2 (2.3%)
Periorbital subcutaneous hematoma	2 (2.3%)
Traumatic iritis	2 (2.3%)
Trochlear nerve palsy	2 (2.3%)
Abducens nerve palsy	1 (1.1%)
Blepharoptosis	1 (1.1%)
Dislocation of intraocular lens	1 (1.1%)
Facial nerve palsy	1 (1.1%)
Nasolacrimal duct obstruction	1 (1.1%)
Oculomotor nerve palsy	1 (1.1%)
Retinal tear	1 (1.1%)
Traumatic angle recession	1 (1.1%)
Traumatic optic neuropathy	1 (1.1%)
Vitreous opacity	1 (1.1%)
Zygomatic and maxillary sinus fracture	1 (1.1%)

Observation was the most frequently performed treatment (Table 3). Eight (9.1%) eyes required ophthalmological treatment or surgery. There were no significant between-group differences in the performed treatments.

**Table 3:** Treatment of ocular injury following traffic accidents The data are presented as N (%). Statistical significance was set at P < 0.05.

	All	Better group	Worse group	P value
Total	88 (100%)	68 (100%)	20 (100%)	-
Observation, n, (%)	54 (61.4%)	41 (60.3%)	13 (65.0%)	0.704
Topical instillation, n, (%)	15 (17.0%)	12 (17.6%)	3 (15.0%)	0.782
Spectacle correction, n, (%)	4 (4.5%)	4 (5.9%)	0 (0.0%)	0.570
Ophthalmological treatment/surgery n, (%)	8 (9.1%)	6 (8.8%)	2 (10.0%)	> 0.999
Others, n, (%)	7 (8.0%)	5 (7.4%)	2 (10.0%)	0.701

Patients with poor visual acuity at the initial examination had significantly worse final visual acuity (P < 0.001) (Table 4). No patient presented with a progression of traumatic cataracts or corneal scarring.

**Table 4:** Ocular presentation at the final visit The data are presented as N (%) or mean ± standard deviation. Statistical significance was set at P < 0.05.

	All	Better group	Worse group	P value
Traumatic cataract, n (%)	0 (0%)	0 (0%)	0 (0%)	> 0.999
Corneal scar, n, (%)	0 (0%)	0 (0%)	0 (0%)	> 0.999
Final CDVA (logMAR)	-0.038 ± 0.35	-0.113 ± 0.079	0.289 ± 0.753	< 0.001
Final intraocular pressure (mmHg)	14.3 ± 3.5	14.3 ± 3.2	14.1 ± 4.8	0.862
Ocular Trauma Score	99.9±1.2	100.0±0.0	99.5±2.5	0.227

The multiple-regression analysis demonstrated that the baseline CDVA was significantly correlated with the final visual acuity (P < 0.001, Table 5).

**Table 5:** Multiple regression analysis to clarify factors to affect final visual acuity Statistical significance was set at P < 0.05. CDVA: corrected-distance visual acuity

	Partial regression coefficients	Standardized partial regression coefficient	P value
Days from injury to the visit (days)	0.000	0.015	0.691
Age (years)	0.001	0.036	0.379
Initial CDVA (logMAR)	1.375	0.933	< 0.001
Availability of referral letters	-0.024	-0.033	0.395
Constant values	0.015	-	0.679

## Discussion

This study examined the ocular prognosis of patients from traffic accidents and analyzed factors related to the final visual acuity in a specialized eye hospital without an emergency department. The included patients had been initially treated in emergency hospitals or clinics in life-threatening conditions and visited our hospital after their general conditions became stable. All the included patients demonstrated relatively good visual acuity (logMAR, −0.038 ± 0.35). This unusually good result could partly be due to the lack of cases of open ocular trauma, which require urgent surgical treatment (e.g., scleral sutures or vitrectomy) and have a poor prognosis [8,11,20]. However, we included one patient with traumatic optic neuropathy secondary to an optic canal fracture, who presented with a loss of vision that did not improve after treatment. Optic nerve or canal injury may cause blindness in 2-5% of patients with facial trauma [21]. Our findings suggest that some patients with mild ocular injury may have serious visual dysfunction. Therefore, thorough examination and prompt judgment regarding the necessity of treatment are required.

Since traffic accidents cause high-energy trauma, the eyes are subjected to strong external forces. Among traffic accident patients with facial injuries, 81.2% present with fractures of the face and nasal bones [22]. Ocular complications associated with traffic accidents can occur secondary to orbital wall fractures. Consistent with previous findings, our patients presented with traumatic damage, including orbital blowout fracture, trochlear nerve palsy, abducens nerve palsy, facial nerve palsy, oculomotor nerve palsy, traumatic optic neuropathy, and zygomatic and maxillary sinus fractures [23,24]. Since these diseases do not necessarily contribute to a direct decline in CDVA, it was difficult to evaluate the abnormalities quantitatively. Specifically, in patients with complaints of diplopia resulting from such injuries, it was difficult to determine the severity of the ocular motility disorder using the data collected in the present study. Nevertheless, some of these patients were appropriately triaged, followed by surgical interventions or prescriptions for new glasses. There has been considerable clinical attention to orbital blowout fractures, which represent a trauma type prone to sequelae [25]. The timing of surgery should be determined based on the patient’s general condition. Moreover, prompt consultation should be performed in a facility capable of conducting surgery.

In our study, the worse group showed significantly worse final visual acuity than the better group. In addition, multivariate linear regression analysis revealed that the final visual acuity was associated with the initial visual acuity, which is consistent with a previous report [8]. Therefore, measuring visual acuity at the initial visit is useful for predicting the long-term prognosis of patients. Traumatic cataracts or corneal scarring after traffic trauma may affect visual outcomes and the prognosis. However, none of our patients presented with these conditions [26]. Nevertheless, the patients we included exhibited various ocular disorders in the anterior segment. These included dry eye exacerbation, abnormal conjunctival tissue resulting from car glass injuries, subconjunctival hemorrhage, and iritis. However, these disorders are relatively mild and do not affect outcomes. It is important to consider ocular posterior segment disorders carefully; moreover, orbital diseases should not be overlooked.

The most frequent diagnosis was globe contusion, which is a representative diagnosis of blunt ocular trauma. The management of globe contusion involves cooling and rest. Moreover, once the eyelid swelling subsides, the effect on visual function is expected to be minimal. Cases of airbag-related ocular injuries have been reported [27]. Notably, this is a complex traumatic condition since it could be associated with various ocular disorders. Moreover, eyelid swelling, with or without subcutaneous hemorrhage, impedes the identification of other anterior and posterior disorders during the initial examination. Ocular trauma can be classified as open or closed-globe injury [28]. In our study, one patient presented with a retinal tear that required photocoagulation. Careful post-treatment observation is essential since retinal detachment or proliferative vitreous retinopathy after vitreous hemorrhage might gradually appear later, even in the absence of abnormalities upon initial examination.

This study had several limitations. First, we could not determine the types and degrees of traffic accidents. Specifically, all patients were referred from other hospitals or visited at their request. Therefore, we could not determine whether the patients were the responsible party, injured party, pedestrians, or drivers in traffic accidents. These differences could have influenced the degrees of high-energy ocular injuries and ophthalmologic prognoses. Further studies are warranted to classify and analyze patients according to the degree of external forces or types of traffic accidents, including those caused by motorbikes or cars. Second, this study was only conducted in a single tertiary hospital in a suburban area, which limits the generalizability of our findings across regions. Therefore, multi-center studies are warranted for further analysis, and a permanent on-call rota for an ophthalmologist in accident and emergency department is important.

## Conclusions

In our research, all the patients demonstrated good visual acuity (logMAR, -0.038 ± 0.35) on average, but some patients with mild ocular injury could result in serious visual dysfunction. Multivariate linear regression analysis revealed that the final visual acuity was associated with the initial visual acuity. Therefore, thorough examination and prompt judgment regarding the necessity of treatment at the initial presentation are essential.
